# Glanders

**Published:** 1883-10

**Authors:** 


### EDITORIAL DEPARTMENT.

### GLANDERS.

THIS formidable disease has been prevalent lately among the horses throughout the State of Illinois with disastrous results, the contagion extending in many instances with fatality to human life.

Dr. N. H. Paaren, State Veterinarian, having been specially commissioned under the authority of the Illinois State Board of Health, to visit the infected localities and institute measures for the suppression of the disease, acted promptly and energetically, doing all in his power to check its extension.

He was resisted, however, in the performance of his duty, it being claimed that he was exercising power not granted him by law in killing the animals affected.

It will be remembered that the Illinois General Assembly in one of its sessions, in consideration of the efforts made under the authority of the Board of Health, amended the so-called "Pleuro-Pneumonia Act" by making its provisions apply also to Glanders. This law declares that horses afflicted with glanders may be slaughtered, whether in an infected district or not, or whether an epidemic exists or not, but in all such cases the surgeon must be backed by the order of at least one of the consulting, or veterinary, or practicing physicians.

The attorneys of the owners contended that Dr. Paaren could not resort to the extreme measure of slaughtering the affected animals until the Governor had issued a proclamation declaring the glanders epidemic. Now, according to the opinion expressed by the Attorney-General, the law requires the proclamation of the Governor to the effect that a district is infected with glanders necessary to the enforcement of quarantine, but as regards authority to slaughter no such proclamation is necessary, only the endorsement by other physicians. If, therefore, Dr. Paaren had such endorsement his action in the matter was perfectly legal.

The Illinois Legislature is to be commended for putting in force the power of the State to prevent the spread of infectious diseases, not only from a financial, but from a humanitarian point of view, since thereby are saved a number of valuable animals, much suffering prevented, both in man and animals, and many human lives preserved. It is to be hoped that the other States in which the disease exists will not be slow to follow the example herein given.

